# Association of Child Maltreatment with South African Adults’ Wages: Evidence from the Cape Area Panel Study

**DOI:** 10.1186/s13561-018-0206-6

**Published:** 2018-09-07

**Authors:** Xiaodong Zheng, Xiangming Fang, Deborah A. Fry, Gary Ganz, Tabitha Casey, Celia Hsiao, Catherine L. Ward

**Affiliations:** 1 College of Economics and Management, China Agricultural University, Beijing, China; 2 School of Public Health, Georgia State University, 140 Decatur Street, Atlanta, GA 30303 USA; 3 Moray House School of Education, University of Edinburgh, Edinburgh, Scotland; 4 Department of Psychology, University of Cape Town, Cape Town, South Africa; 5 Save the Children South Africa, Johannesburg, South Africa

**Keywords:** Child maltreatment, Physical abuse, Emotional abuse, Wages, Heckman selection model, South Africa, I18, J30, J31

## Abstract

Child maltreatment is a prevalent public health problem in both developed and developing countries. While many studies have investigated the relationship between violence against children and health of the victims, little is known about the long term economic consequences of child maltreatment, especially in developing countries. Using data from the Cape Area Panel Study, this paper applies Heckman selection models to investigate the relationship between childhood maltreatment and young adults’ wages in South Africa. The results show that, on average, any experience of physical or emotional abuse during childhood is associated with a later 12% loss of young adults’ wages. In addition, the correlation between physical abuse and economic consequence (14%) is more significant than the relationship between emotional abuse and wages (8%) of young adults; and the higher the frequency of maltreatment, the greater the associations with wages. With respect to gender differences, wage loss due to the experience of childhood maltreatment is larger for females than males. Specifically, males’ wages are more sensitive to childhood emotional abuse, while females’ wages are more likely to be affected by childhood physical abuse. These results emphasize the importance of prioritizing investments in prevention and intervention programs to reduce the prevalence of child maltreatment and to help victims better overcome the long-term negative effect.

## Background

Child maltreatment remains a prevalent public health problem in both developed and developing countries. In South Africa, violence against children, abuse, and neglect are widespread. According to the household survey of the Optimus Study South Africa, a nationally representative study of 15–17-year olds, 18% had experienced physical abuse and 26.1% emotional abuse, in their lifetimes [1]. Across Africa, more than half of children have experienced maltreatment in their lifetime, and more than one fourth report lifetime multiple abuse victimization [2, 3]. There is increasing evidence that child maltreatment is associated with serious consequences for child development, including mental health [4–7], physical health [8–10], and academic, social, and behavioral functioning [11–14]. Little is known, however, about the association between childhood maltreatment and wages of adults, especially in developing countries. The purpose of this paper is therefore to explore the long-term consequences of child maltreatment on adulthood wages in South Africa.

Evidence of the link between child maltreatment and economic consequences is important for several reasons. First, the long-term consequences of child maltreatment on adult wages should improve the understanding of the economic burden of child maltreatment to society [15–17]. Second, the lifelong economic consequences of child maltreatment provide another key element for the comprehension of the formation of inequality and its persistence. Evidence shows that socioeconomic status is an important risk factor for child maltreatment [18–20]. If child maltreatment is associated with later adverse socioeconomic attainment, then it would be crucially linked to income and class inequality [21]. Third, estimating the variation in adult wages caused by childhood maltreatment will help policymakers and government officials prioritize funding and develop services to reduce or prevent child maltreatment.

Child maltreatment can cause gross physical trauma to the brain, including hypothalamic-pituitary-adrenal axis dysregulation, as well as parasympathetic and catecholamine responses [22, 23]. Therefore, it is not surprising that child maltreatment leads to consequences such as adverse mental health, physical health, and educational outcomes [24, 25]. These consequences may in turn have an effect on later economic productivity and socioeconomic attainment of the victims [26, 27].

Several studies used data from developed countries such as the USA and New Zealand to investigate this issue. Among them, Macmillan examined the relationship between adolescent victimization, which can broadly be understood as a form of child maltreatment, and later income in adulthood [28]. The results indicated that adolescent violent victimization reduced hourly income by an average of 14%. Mullen and colleagues and Hyman studied the socioeconomic consequences of childhood sexual abuse [29, 30]. They found that childhood sexual abuse had a significant adverse effect on the earnings of women. Zielinski found that adults who had experienced maltreatment in childhood had significantly higher rates of unemployment, poverty, and Medicaid usage in the USA [27]. Currie and Widom applied a prospective cohort design to examine the adult economic status and productivity consequences of early victimization of child maltreatment, and found that individuals with histories of maltreatment had lower levels of education, employment, and earnings, and fewer assets in middle age [26].

Several studies from South Africa have shown associations between child maltreatment with psychosocial and educational outcomes as well as quality of life [1, 31–33]. However, to date very few studies have focused on the relationship between childhood maltreatment and economic well-being of adults in developing countries like South Africa.

Using the Heckman Selection Model to overcome problems of self-selection bias, this study empirically examined the association of maltreatment during childhood with adult wages in South Africa. Because previous studies indicated that child maltreatment was more detrimental to the health and educational outcomes of girls [34–37] and frequency of abuse was an important marker of severity [38, 39], which may affect subsequent economic consequences, we also investigated the the impact of gender and frequency of maltreatment on the relationship between child maltreatment and young adults’ wages.

## Methods

### Data

The data used in this study is from the Cape Area Panel Study (CAPS). CAPS is a longitudinal study that follows the lives of a representative sample of youth and young adults as they undergo the multiple transitions from adolescence to adulthood in Cape Town, South Africa. The study commenced in 2002 as a collaborative project of the Universities of Cape Town and Michigan. The CAPS household sample was drawn through a two-stage process. First, a sample of primary sampling units (PSUs) was selected within each population group stratum with probability proportional to size. Second, a sample of 25 screener households was drawn within each PSU, and the adolescents aged 14–22 in each selected family were the respondents in Wave 1 [40]. Currently, CAPS includes five waves from 2002 to 2009 [41] and the dataset has been increasingly used in recent years to examine issues related to education, employment, and health of youth and young adults in South Africa [42–46].

We used two sets of indicators, from Waves 1 and 5. Child maltreatment indicators are included in Wave 1, in which young adults aged 14–22 were asked to recall their childhood abuse; and socioeconomic outcomes in Wave 5 when the age of young adults was 21–29. Wave 1 of CAPS successfully interviewed 4752 youth or young adults in face-to-face interviews, and Wave 5 successfully followed 2915 respondents [41]. Because of missing data in Wave 5, the final sample size in our study was 2644. Bivariate analyses across gender, age, race, education levels and marital status were conducted to examine the differences between excluded missing cases in Wave 5 and non-missing cases, and no significant differences were found. In addition to unweighted data, we also used weighted CAPS data to provide results that were based on a more reasonably representative sample. The weighted distribution of participants was generated in line with population group distribution in Cape Town, which was within one percentage point bias from the 1996 census [40].

### Measures

#### Child maltreatment

The key dependent variables of physical and emotional abuse used in this study are retrospective questions about child maltreatment from the first wave of CAPS in 2002. Respondents were asked to reflect on their family life up until they were 14 years of age. The questions with regard to child maltreatment, which were adapted from the Conflict Tactics Scale (CTS) [47], included how often a perpetrator had sworn at or insulted them, or put them down (“put down”); made them afraid that they might be physically hurt (“afraid of hurt”); pushed, grabbed, slapped, or thrown something at them (“push”); and hit them so hard that they had marks or were injured (“hit hard”). The respondents were also asked whether a parent, stepparent, or an adult living in their home was the perpetrator. Following Chapman et al. and Dube et al. [48, 49], “put down” and “afraid of hurt” are considered as emotional abuse, while “push” and “hit hard” are regarded as physical abuse. The respondents were asked to report the frequency of maltreatment on a five-point Likert scale: never, once or twice, sometimes, often, very often.

From each answer to the questions relevant with child maltreatment, we generated two types of child maltreatment variables. The first type was a dummy variable which was scored 0 if the respondent answered “never,” otherwise scored 1. The second type was composed of two dummy variables: whether the respondent answered “once or twice” or “sometimes” (low frequency maltreatment), and whether the respondent answered “often” or “very often” (high frequency maltreatment). Type 1 indicates whether the respondent has been maltreated or not, whereas Type 2 divides the respondents into three categories (non-maltreated, low frequency maltreatment, and high frequency maltreatment). In order to differentiate the association of physical abuse and emotional abuse with young adults’ wages, we generated dummy variables for “emotional abuse”: scored 1 if the young adult had once suffered from “put down” or “afraid of hurt,” otherwise scored 0; “physical abuse”: scored 1 if the young adult had once suffered from “push” or “hit hard”, otherwise scored 0; and “any child maltreatment”: scored 1 if the respondent had suffered from any kind of maltreatment in childhood, scored 0 if the respondent had never been maltreated.

Although the retrospective measures of child maltreatment can almost rule out the possibility of reverse causation [50], several biases are possible. First, recall bias exists if young adults forget childhood maltreatment or do not recognize what they experienced as a child as maltreatment. Second, reporting bias may exist if young adults choose not to disclose such private information; this may particularly be true of males [1]. Third, selection bias may occur if young adults refuse to answer the question. Pieterse used the same data to test the possible biases and found that recall bias and selection bias were not a serious problem [25]. Although females are more likely to report experiences of childhood maltreatment, there was no evidence to determine whether that was reporting bias or a genuine gender difference. Other studies have shown that the childhood maltreatment CAPS data is properly collected and thus provides a valid and reliable measure of maltreatment in childhood [48, 49, 51].

#### Economic outcome

The respondent’s monthly wages (measured in South African Rands, ZAR) in the fifth wave of CAPS in 2009, were used as the economic outcome variable. Young adults were asked to report take-home pay after taxes and other deductions in a typical month.

#### Control variables

Various factors are associated with the wages of young adults, including personal and household characteristics. Omitting variables which are both correlated with child maltreatment and adulthood wages would bias estimates in the regression models. Since family environment in childhood may both be correlated with adulthood wages and an important determinant of child maltreatment, we used childhood household characteristics in Wave 1 rather than that in Wave 5 to reduce the possibility of endogeneity problems as far as possible. Therefore, in the empirical analyses, we controlled for individual characteristics from Wave 5 and household characteristics from Wave 1. The controls included gender, race, age, age squared, education levels, marital status, home language, household size, the gender of household head, mother’s education (and also whether an observation of mother’s education was missing) and the family income per capita. Compared to existing relevant studies [25–27], this study controlled more relevant variables, especially childhood household characteristics.

The measures of variables used in this study were summarized in Appendices A1 (available at https://drive.google.com/open?id=1TW54hbQ7dt4PKQ86bwfd2p8hC5ZJRlWP).

### Descriptive analyses

The descriptive statistics for childhood maltreatment of young respondents in the CAPS are reported in Table 1, with *t*-tests for different groups by gender and race. The overall prevalence of child maltreatment in the sample was 59%: 34% of young adults reported having been physically abused, and 54% having been emotionally abused. From *t*-test results, a significantly higher proportion of females have been “put down” and “afraid of hurt” by adults compared to males. The prevalence of child maltreatment of Colored youth is significantly higher than that of Black adolescents.

**Table 1:** Childhood maltreatment by gender and race (unweighted)

Variable	Total (*N* = 2644)	Gender				Race		
		Female (*N* = 1447)	Male (*N* = 1197)	*t*-test		Black African (*N* = 1219)	Colored (*N* = 1310)	*t*-test
Put down	0.49	0.51	0.47	1.80^*^		0.42	0.57	−7.77^***^
Afraid of hurt	0.29	0.30	0.27	1.96^**^		0.32	0.27	3.18^***^
Push	0.31	0.31	0.32	−0.20		0.24	0.39	−8.09^***^
Hit hard	0.12	0.12	0.13	−0.07		0.09	0.16	−5.48^***^
Put down (high)	0.08	0.09	0.07	2.47^**^		0.04	0.12	−7.73^***^
Afraid of hurt (high)	0.04	0.04	0.03	1.42		0.02	0.06	−3.97^***^
Push (high)	0.05	0.05	0.04	0.92		0.02	0.07	−5.60^***^
Hit hard (high)	0.02	0.02	0.02	−0.97		0.01	0.03	−2.15^**^
Physical abuse	0.34	0.34	0.34	0.16		0.26	0.42	−8.56^***^
Emotional abuse	0.54	0.55	0.52	1.54		0.47	0.61	−6.92^***^
Any child maltreatment	0.59	0.60	0.57	1.25		0.51	0.66	−7.70^***^

Note: Statistics in *t*-test column are t values. The terms “Black African” and “Colored” date from the Apartheid era in South Africa. Our use of them does not imply support for these racialised categories; rather, we report them because of their continuing association with health and other inequalities [52]. The term Black African means Black people; the terms Colored means mixed-race South Africans. Since there were too few observations of White and Indian peoples, the table does not report statistics for these groups in the “Race” column. “High” refers to high frequency

*** *p* < 0.01, ** *p* < 0.05, * *p* < 0.1

Table 2 presents the monthly wages of the young adults who worked in 2009, and shows *t*-tests for groups by gender and race. The average monthly wages of young adults in the Cape Town area was ZAR 3058.55 (nearly $413 USD) in 2009. Results of *t*-tests show that males earned more than females, and Colored people had higher wages than Black people. Descriptive statistics for respondents’ individual and household characteristics are reported in Table 3. The weighted means show that the adolescents were predominately female (51%), Colored (56%), single (82%) and not well educated (only 14% percent of the sample had graduated from college). 24% of household heads were female and 38% of families’ home language was English. The mean household size and household income per capita were 5.55 and 831.44 ZAR, respectively.

**Table 2:** Young adults’ monthly wage by gender and race (unweighted)

Variable	Total (*N* = 1786)	Gender				Race		
		Female (*N* = 934)	Male (*N* = 852)	*t*-test		African (*N* = 732)	Colored (*N* = 1006)	*t*-test
Monthly wages (ZAR)	3058.55	2929.04	3200.52	−2.55^**^		2326.62	3440.99	−11.69^***^
	(3515)	(2125.06)	(2365.67)			(1567.09)	(2192.43)	

Note: Standard deviation in parentheses

*** *p* < 0.01, ** *p* < 0.05, * *p* < 0.1

**Table 3:** Individual and household characteristics (*N* = 2644)

Variable	Symbol	Mean (sample)	Mean (weighted)	Standard deviation	Wave
Individual Characteristics					
Male	male	0.45	0.49	0.50	Wave 5
African	african	0.46	0.29	0.50	Wave 5
Colored	colored	0.50	0.56	0.50	Wave 5
Age	age	24.47	24.67	2.58	Wave 5
Age square	age_sqr	605.42	615.11	127.54	Wave 5
College degree	coll	0.07	0.12	0.25	Wave 5
College degree above	coll_ab	0.02	0.02	0.13	Wave 5
Married	marr	0.14	0.16	0.35	Wave 5
Separated	sepa	0.02	0.02	0.13	Wave 5
In school	school	0.09	0.11	0.28	Wave 5
Have a child	child	0.33	0.29	0.47	Wave 5
Household Characteristics					
Home language English	hlang	0.16	0.24	0.36	Wave 1
Household size	hsize	5.77	5.55	2.50	Wave 1
Female-headed household	hhead_fe	0.40	0.38	0.12	Wave 1
Mother’s education	mo_edu	0.12	0.10	0.33	Wave 1
Mother’s education missing	mo_edu_miss	0.82	0.85	0.38	Wave 1
Household income per capita (log)	lnfaminc	6.11	6.47	0.02	Wave 1

### Empirical analyses

Our econometric analysis took place in three steps. First, we used a probit model to estimate the probability of adulthood employment as a function of child maltreatment and control variables. Second, we use an ordinary least square (OLS) model to estimate the association of child maltreatment with adulthood wages, as follows:

1 $$ {\displaystyle \begin{array}{c}{lnwage}_i={\beta}_0+{\beta}_1{maltreated}_i+{\beta}_2{male}_i+{\beta}_3{african}_i+{\beta}_4{colored}_i+{\beta}_5{age}_i+{\beta}_6 age\_{sqr}_i\\ {}+{\beta}_7{coll}_i+{\beta}_8 coll\_{ab}_i+{\beta}_9{marr}_i+{\beta}_{10}{sepa}_i+{\beta}_{11}{hlang}_i+{\beta}_{12}{hsize}_i+{\beta}_{13} hhead\_{fe}_i\\ {}+{\beta}_{14} mo\_{edu}_i+{\beta}_{15} mo\_ edu\_{miss}_i+{\beta}_{16}{lnfaminc}_i+{\varepsilon}_{1i}\end{array}} $$

where *lnwage*_*i*_ represents natural logarithm of the *i*th respondent’s monthly wages; and *maltreated* is an indicator for the *i*th respondent who had a substantiated case of a type of maltreatment in childhood; *ɛ* represents the random error term; and *ß*_*i*_, the coefficient of *maltreated*, is the elasticity of wage to child maltreatment.

However, the OLS model does not take into account possible sample selection bias because our study has access to wage observations for only those who work, who are not randomly selected from the population. For example, adult respondents who did not work were more likely to have lower education, to be students, or to have a child, especially for women [53, 54]. Estimating the association of child maltreatment with adulthood wages from subpopulation who work by using OLS regressions may introduce bias. Therefore, in our third phase of econometric analyses we applied Heckman selection models [55–57] to correct for such sample selection bias. The Heckman selection model takes place in two stages: the selection equation, and then and the outcome equation. In the first stage, we formulated a selection equation using probit regression to estimate the probability of working. The extended form of the selection equation is as follows:

2 $$ {\displaystyle \begin{array}{c}\mathrm{Prob}\left({\mathrm{wage}}_{\mathrm{i}}>0\right)={\beta}_0+{\beta}_1{maltreated}_i+{\beta}_2{male}_i+{\beta}_3{african}_i+{\beta}_4{colored}_i+{\beta}_5{age}_i\\ {}+{\beta}_6 age\_{sqr}_i+{\beta}_7{coll}_i+{\beta}_8 coll\_{ab}_i+{\beta}_9{marr}_i+{\beta}_{10}{sepa}_i+{\beta}_{11}{hlang}_i\\ {}+{\beta}_{12}{hsize}_i+{\beta}_{13} hhead\_{fe}_i+{\beta}_{14} mo\_{edu}_i+{\beta}_{15} mo\_ edu\_{miss}_i\\ {}+{\beta}_{16}{lnfaminc}_{i+}{\beta}_{17}{school}_i+{\beta}_{18}{child}_i+{\varepsilon}_{2i}\end{array}} $$

where *school* and *child* are the unique regressors in the selection equation, and.

3 $$ {\varepsilon}_1\sim N\left(0,\sigma \right) $$

4 $$ {\varepsilon}_2\sim N\left(0,1\right) $$

5 $$ corr\ \left({\varepsilon}_{1,}{\varepsilon}_2\right)=\rho $$

where *ρ* represents the correlation of error term *ɛ*_*1*_ and *ɛ*_*2*_. The simple OLS regression technique yields biased results when *ρ ≠ 0*, which demonstrates the observed young adults are not randomly selected. In such circumstance, the Heckman selection model estimates are consistent and asymptotically efficient.

In the second stage, we corrected for self-selection in the outcome equation, which is the same as eq. (1), by incorporating a transformation of the predicted probabilities in the first stage as an additional explanatory variable. We firstly used two types of Heckman selection model: Heckman Two-Step (H2S) procedure, and maximum likelihood estimation (MLE). The non-selection hazard or Inverse Mill’s Ratio (λ) is computed and used as an additional explanatory variable in outcome equation in H2S estimation, while *σ* and *ρ* are indirectly estimated by ln*σ* and atahn*ρ* in the MLE to correct sample selection bias in the outcome equation.

In addition to H2S and MLE, we also used weighted Heckman maximum likelihood estimation (weighted MLE) to check the robustness of the model results.

## Results and discussion

### Different subtypes and frequency of child maltreatment and adulthood wages

Table 4 shows the probit and OLS regression estimates of the relationship between different subtypes (with different frequency levels) of childhood maltreatment and adulthood employment and wages in South Africa. Estimates from the probit models show that any experience of different subtypes of childhood maltreatment is not significantly correlated with the employment status (employment vs. unemployment) of the young adults. However, higher frequency of physical abuse such as “push” is associated with lower probability of working, showing that child maltreatment may also correlate with lower levels of labor supply, which coincides with Zielinski’s study [27]. The OLS regressions were applied only to the subsample of respondents who worked and earned income. All subtypes of child maltreatment except “hit hard” were significantly correlated with lower wages. The estimates show that any experience of “put down”, “afraid of hurt” and “push” were associated with 6%, 8% and 12% loss of young adults’ wages respectively, indicating that the relationship between “push” and economic consequence is stronger. Compared to low frequency of childhood maltreatment, high frequency of childhood violence resulted in more adverse economic situations for young adults.

**Table 4:** Estimates of probit and OLS regressions

Variables	probit: whether at work				OLS: wages			
	Coefficient	Standard error	Coefficient	Standard error	Coefficient	Standard error	Coefficient	Standard error
Put down	0.00	(0.05)			−0.06^**^	(0.03)		
Put down (low)			0.01	(0.05)			−0.04	(0.03)
Put down (high)			− 0.06	(0.10)			−0.14^***^	(0.05)
R^2^ / Pseudo-R^2^	0.059		0.058		0.21		0.21	
Observations	2644		2644		1786		1786	
Afraid of hurt	−0.03	(0.06)			− 0.08^***^	(0.03)		
Afraid of hurt (low)			−0.03	(0.06)			−0.08^**^	(0.03)
Afraid of hurt (high)			−0.07	(0.13)			−0.14^*^	(0.07)
R^2^ / Pseudo-R^2^	0.059		0.058		0.21		0.21	
Observations	2644		2644		1786		1786	
Push	−0.06	(0.05)			− 0.12^***^	(0.03)		
Push (low)			−0.02	(0.06)			−0.11^***^	(0.03)
Push (high)			−0.26^**^	(0.13)			−0.24^***^	(0.07)
R^2^ / Pseudo-R^2^	0.059		0.060		0.21		0.21	
Observations	2644		2644		1786		1786	
Hit hard	−0.05	(0.08)			− 0.06	(0.04)		
Hit hard (low)			−0.02	(0.08)			−0.05	(0.04)
Hit hard (high)			−0.22	(0.19)			−0.16^*^	(0.10)
R^2^ / Pseudo-R^2^	0.059		0.059		0.21		0.21	
Observations	2644		2644		1786		1786	

Note: Coefficients for each subtype of child maltreatment come from separated regressions. In both models, the following controls were included: gender, race, age, age squared, education level, marital status, home language, household size, female-headed household, mother’s education, and household per capita income. Standard error in parentheses

*** *p* < 0.01, ** *p* < 0.05, * *p* < 0.1

Table 5 presents the results of Heckman selection models with different subtypes and frequency of child maltreatment. To economise on space, we only report the estimates of the coefficients of the child maltreatment variables. The full information estimation results of Heckman selection models are presented in Appendices A2 to A10 (available at https://drive.google.com/open?id=1TW54hbQ7dt4PKQ86bwfd2p8hC5ZJRlWP). In all cases, the selective terms (atahn*ρ*, ln*σ*) are statistically significant, which shows that the estimates in OLS regressions are biased and the application of the Heckman selection model is the more reasonable and reliable approach. The results in the left side of Table 5 show that all measures of child maltreatment are significant except “hit hard” in the MLE column. The estimates of child maltreatment in the three types of Heckman selection model are relatively close. The results of weighted MLE estimation indicate that any experience of physical abuse (“push” or “hit hard”) in childhood is associated with more adverse economic consequences than emotional abuse (“put down” or “afraid of hurt”). Overall, “put down”, “afraid of hurt”, “push” and “hit hard” are associated with 8%, 10%, 14% and 7% wages loss, implying that the estimates in OLS regressions are underestimated. In addition, low frequency and high frequency child maltreatment are associated with 7% to 13%, and 15% to 25%, more loss of wages than that of young adults who have never been abused in childhood, which also demonstrates that the higher frequency of being maltreated in childhood is associated with more negative economic earning ability in adulthood.

**Table 5:** Estimates of association between each subtype of child maltreatment and adulthood wages (N = 2644)

Variables	H2S		MLE		Weighted MLE	
	Coefficient	Standard error	Coefficient	Standard error	Coefficient	Standard error
Child maltreatment: never vs. at least once						
Put down	−0.08^**^	(0.03)	− 0.06^**^	(0.03)	− 0.08^**^	(0.03)
Afraid of hurt	−0.10^***^	(0.03)	−0.08^***^	(0.03)	−0.09^**^	(0.04)
Push	−0.14^***^	(0.03)	−0.13^***^	(0.03)	−0.15^***^	(0.03)
Hit hard	−0.07^*^	(0.04)	−0.06	(0.04)	−0.12^***^	(0.05)
Child maltreatment: never vs. low frequency; never vs. high frequency						
Put down (low)	−0.07^*^	(0.03)	−0.05	(0.03)	−0.06	(0.04)
Put down (high)	−0.16^***^	(0.06)	−0.14^***^	(0.05)	−0.16^**^	(0.06)
Afraid of hurt (low)	−0.09^***^	(0.03)	−0.08^**^	(0.03)	−0.08^**^	(0.04)
Afraid of hurt (high)	−0.15^**^	(0.08)	−0.14^*^	(0.07)	−0.20^**^	(0.08)
Push (low)	−0.13^***^	(0.04)	−0.12^***^	(0.03)	−0.14^***^	(0.04)
Push (high)	−0.25^***^	(0.08)	−0.24^***^	(0.07)	−0.27^***^	(0.06)
Hit hard (low)	−0.06	(0.05)	−0.05	(0.04)	−0.10^*^	(0.05)
Hit hard (high)	−0.17^*^	(0.10)	−0.16^*^	(0.10)	−0.22^**^	(0.10)

*Note*: Coefficients for each subtype of child maltreatment come from separated regressions. Regressions in Heckman outocme equation with controls including gender, race, age, age squared, education level, marital status, home language, household size, female-headed household, mother’s education and household per capita income. Controls in Heckman selection equation are the same elements plus “have a child” and “in school”. “low” and “high” refer to low frequency and high frequency respectively. Standard error in parentheses

*** *p* < 0.01, ** *p* < 0.05, * *p* < 0.1

Table 6 reports the Heckman selection model estimates of the associations of overall physical abuse, emotional abuse, and any maltreatment in childhood with wages of young adults. The selectivity terms in all cases, and each coefficient of child maltreatment in the Heckman selection model, are also significant, and the results in three types of Heckman selection model are almost the same. The estimates show that, compared to those respondents who did not report the corresponding type of child maltreatment, having experience of physical abuse, emotional abuse, and any child maltreatment are associated with 12%–14%, 7%–9%, and 11%–13% loss of wages respectively, which also shows that the long-term negative economic consequences of physical abuse are more severe than the effect resulting from emotional abuse. This result is consistent with related studies [26, 27]. Previous studies suggest that physical abuse is particularly associated with behavior problems, marital breakdown, and low educational levels, while emotional abuse is correlated with low self-esteem and psychological distress [25, 29, 58, 59]. The more adverse social, educational and health consequences of physical abuse may explain why physical abuse in childhood is more detrimental to the later economic consequences than emotional abuse.

**Table 6:** Estimates of association between child maltreatment and adulthood wages (N = 2644)

Variables	H2S		MLE		Weighted MLE	
	Coefficient	Standard error	Coefficient	Standard error	Coefficient	Standard error
Physical abuse	−0.13^***^	(0.03)	−0.12^***^	(0.03)	−0.14^***^	(0.03)
Emotional abuse	−0.09^***^	(0.03)	−0.07^**^	(0.03)	−0.08^**^	(0.04)
Any child maltreatment	−0.13^***^	(0.04)	−0.11^***^	(0.03)	−0.12^***^	(0.04)

*Note*: Coefficients for each subtype of child maltreatment come from separated regressions. Regressions in Heckman outocme equation with controls including gender, race, age, age squared, education level, marital status, home language, household size, female-headed household, mother’s education and household per capita income. Controls in Heckman selection equation are the same elements plus “have a child” and “in school”. “low” and “high” refer to low frequency and high frequency respectively. Standard error in parentheses

*** *p* < 0.01, ** *p* < 0.05, * *p* < 0.1

### Child maltreatment and adulthood wages: Gender differences

Table 7 reports the estimates of Heckman selection models by gender, which indicates the relationship between different subtypes of childhood maltreatment and young adults’ wages. Overall, the negative correlations between all subtypes of childhood maltreatment and females’ wages are higher. The associations between “put down” and “afraid of hurt” and the wages of male adults are not significant, while they are significant for females. Both “push” and “hit hard” in childhood are significantly negative for both male and female young adults. On average, as shown in the estimates of the weighted MLE models, the proportion of wage loss for young female adults is, respectively, 6%, 4%, 12%, and 4% higher than males, for any experience of being put down, afraid of being hurt, pushed and hit hard.

**Table 7:** Estimates of association between each subtype of child maltreatment and adulthood wages: gender difference

Variables	H2S		MLE		Weighted MLE	
	Coefficient	Standard error	Coefficient	Standard error	Coefficient	Standard error
Male (*N* = 1197)						
Put down	−0.06	(0.04)	−0.06	(0.04)	−0.05	(0.05)
Afraid of hurt	−0.06	(0.04)	−0.06	(0.04)	−0.07	(0.05)
Push	−0.08^*^	(0.04)	−0.07^*^	(0.04)	−0.09^**^	(0.04)
Hit hard	−0.06	(0.06)	−0.05	(0.06)	−0.09	(0.07)
Physical abuse	−0.07^*^	(0.04)	−0.07^*^	(0.04)	−0.08^*^	(0.05)
Emotional abuse	−0.05	(0.04)	−0.05	(0.04)	−0.03	(0.05)
Any child maltreatment	−0.07	(0.04)	−0.06	(0.04)	−0.06	(0.05)
Female (*N* = 1447)						
Put down	−0.10^*^	(0.05)	−0.06	(0.04)	−0.11^**^	(0.05)
Afraid of hurt	−0.13^***^	(0.05)	−0.10^**^	(0.04)	−0.11	(0.07)
Push	−0.20^***^	(0.05)	−0.18^***^	(0.04)	−0.21^***^	(0.05)
Hit hard	−0.08	(0.06)	−0.07	(0.06)	−0.13^*^	(0.07)
Physical abuse	−0.19^***^	(0.05)	−0.17^***^	(0.04)	−0.20^***^	(0.05)
Emotional abuse	−0.14^**^	(0.06)	−0.09^**^	(0.04)	−0.14^**^	(0.06)
Any child maltreatment	−0.21^**^	(0.09)	−0.15^***^	(0.04)	−0.19^***^	(0.06)

*Note*: Coefficients for each subtype of child maltreatment come from separated regressions. Regressions in Heckman outocme equation with controls including gender, race, age, age squared, education level, marital status, home language, household size, female-headed household, mother’s education and household per capita income. Controls in Heckman selection equation are the same elements plus “have a child” and “in school”. “low” and “high” refer to low frequency and high frequency respectively. Standard error in parentheses

*** *p* < 0.01, ** *p* < 0.05, * *p* < 0.1

Table 7 also presents the estimates of the associations of overall physical abuse, emotional abuse and child maltreatment with young adults’ wages. For males, estimates show that physical abuse is more significantly associated with lower wages in adulthood, while emotional abuse and overall maltreatment are less strongly correlated with wages. For females, all the coefficients of physical abuse, emotional abuse, and overall child maltreatment are significantly negative. Specifically, experiencing physical abuse, emotional abuse and overall child maltreatment are associated with 8%, 3% and 6% loss of young male adults’ monthly wages respectively, and 20%, 14%, 19% loss of wages respectively for females. In other words, the proportion of wages lost from any experience of the three types of child maltreatment is more than twice as great for females as for males. While many studies exploring the consequences of sexual abuse control for gender rather than exploring outcomes by gender, some studies do find risks that may explain this. For instance, compared to males, females who have been maltreated in childhood are at increased risk of alcohol abuse and health problems [26, 60]. A global systematic review and meta-analysis has also found that child maltreatment impacts differentially on boys and girls in terms of educational absenteeism, which may have subsequent impact on employment and wage earnings [34]. However, other studies find that, while genders may differ in the kind of outcome they experience (for instance, females are more likely to experience internalizing disorders and males externalizing disorders), both kinds of outcome are likely to affect earning capacity [24]. Future studies should explore the variables that mediate and moderate the relationship between child maltreatment and adult wages.

Table 8 presents the three types of Heckman selection model estimates of the associations of different frequency of child maltreatment with young adults’ wages. Estimation results indicate that high frequency of “afraid of hurt,” on the one hand, is more significantly correlated with lower wages of young male adults, compared to young female adults. On the other hand, the proportion of wage loss for females is higher than that of young male adults for the high frequency of “push” and “hit hard.” We also find the coefficients of low frequency and high frequency “afraid of hurt” for males and “hit hard” for females are greatly different, which implies that there may be a big variation of the effect between different frequencies of child maltreatment. Future studies should explore differences in effect between long-term and frequent child maltreatment, and short-term, less frequent maltreatment.

**Table 8:** Estimates of association between different frequency of each subtype of child maltreatment and adulthood wages: gender difference

Variables	H2S		MLE		Weighted MLE	
	Coefficient	Standard error	Coefficient	Standard error	Coefficient	Standard error
Male (*N* = 1197)						
Put down (low)	−0.04	(0.04)	−0.04	(0.04)	−0.03	(0.05)
Put down (high)	−0.17^**^	(0.08)	−0.16^**^	(0.08)	−0.14^*^	(0.08)
Afraid of hurt (low)	−0.04	(0.05)	−0.03	(0.05)	−0.03	(0.05)
Afraid of hurt (high)	−0.23^**^	(0.11)	−0.23^**^	(0.11)	−0.30^***^	(0.11)
Push (low)	−0.07	(0.04)	−0.06	(0.04)	−0.09^*^	(0.05)
Push (high)	−0.18^*^	(0.10)	−0.17^*^	(0.10)	−0.19	(0.12)
Hit hard (low)	−0.06	(0.06)	−0.05	(0.06)	−0.09	(0.08)
Hit hard (high)	−0.02	(0.13)	−0.01	(0.13)	−0.10	(0.16)
Female (*N* = 1447)						
Put down (low)	−0.09	(0.06)	−0.04	(0.04)	−0.09^*^	(0.05)
Put down (high)	−0.16^*^	(0.09)	−0.11	(0.07)	−0.17^*^	(0.09)
Afraid of hurt (low)	−0.13^**^	(0.06)	−0.10^**^	(0.05)	−0.12^**^	(0.06)
Afraid of hurt (high)	−0.12	(0.11)	−0.10	(0.10)	−0.14	(0.11)
Push (low)	−0.20^***^	(0.06)	−0.17^***^	(0.04)	−0.19^***^	(0.05)
Push (high)	−0.30^**^	(0.13)	−0.31^***^	(0.09)	−0.35^***^	(0.06)
Hit hard (low)	−0.05	(0.07)	−0.04	(0.06)	−0.10	(0.07)
Hit hard (high)	−0.27^*^	(0.14)	−0.28^**^	(0.14)	−0.29^***^	(0.09)

*Note*: Coefficients for each subtype of child maltreatment come from separated regressions. Regressions in Heckman outocme equation with controls including gender, race, age, age squared, education level, marital status, home language, household size, female-headed household, mother’s education and household per capita income. Controls in Heckman selection equation are the same elements plus “have a child” and “in school”. “low” and “high” refer to low frequency and high frequency respectively. Standard error in parentheses

*** *p* < 0.01, ** *p* < 0.05, * *p* < 0.1

## Conclusion and policy implications

Using data from the Cape Area Panel Study (CAPS), this study applied three types of Heckman selection model, including Heckman Two-Step estimation, and weighted and unweighted Heckman maximum likelihood estimation, to study the association of child maltreatment with young adults’ monthly wages in South Africa. The results are consistent across the three types of model: on average, compared to an individual who has no history of childhood maltreatment, any experience of child maltreatment is associated with a 12% loss of young adults’ wages. At the same time, physical abuse is more strongly correlated with adverse economic consequence than emotional abuse, which coincides with Zielinski’s findings [27]. In addition, young adults appear to be more strongly affected by high frequency childhood maltreatment than low frequency childhood maltreatment.

Consistent with some studies (e.g., [26]), but not with others (e.g., [24]), we identified gender differences in the association of child maltreatment with wages. Females were more strongly affected by the experience of childhood maltreatment than males. Also, compared to young female adults, high-frequency emotional abuse had a more severe adverse impact on the wages of young male adults, while the proportion of wages lost for females was higher than that of males for high frequency physical abuse. Future research should explore whether our findings are replicated in other samples and contexts, given that some studies find otherwise. The mechanisms by which this relationship comes about should also be explored in future research, so as to inform the development and implementation of prevention and intervention programs that are gender-sensitive. In addition, we also encourage future studies to investigate the possible relationship between child maltreatment and economic inequality, which would help to expand our knowledge of the effect of violence against children.

Our findings also bear significant policy implications. First, implementing support programs for parents, teachers, and other caregivers that provide alternatives to violent punishment and prevent child maltreatment, is an urgent need. In the area of child maltreatment, parent training programs including parent-infant home-visiting programs and group parent-training programs for parents of older children, have demonstrated effectiveness for reducing child maltreatment in other contexts [61, 62]. Importantly, several parent training programs have demonstrated cost-effectiveness for reducing child maltreatment in high-income countries such as the USA and are showing promise in South Africa [63, 64]. Such programs should urgently be introduced into low- and middle-income contexts, such as South Africa, and their cost-effectiveness investigated in the new contexts, as a tool for advocating with governments for their use. Recommendations feeding into such plans must be based on sound evidence, and will only be implementable if adequately resourced. Evidence from this study can contribute to building a strong case for state funding of violence prevention programs to be prioritized.

Second, a national policy, which aims at providing sufficient, subsidised healthcare services to children at risk and special education and rehabilitation services for victims of maltreatment, is urgently required, in order to assist victims of maltreatment to overcome the mental and physical health consequences of such abuse.

There are several limitations in this study. First, there may be measurement error in the data on maltreatment and, since the prevalence of maltreatment was reported in retrospect, it could be underreported. This underreporting could results from unwillingness to disclose such private information in an interview, but it is also possible that respondents have repressed traumatic memories or do not recognize that what they experienced as a child was actually maltreatment. The difference between actual maltreatment and disclosed maltreatment may cause underestimation of the association between childhood maltreatment and adults’ wages in South Africa. Second, because the CAPS does not provide information on sexual abuse and neglect, which also constitute forms of child maltreatment, this study was unable to investigate these forms of child maltreatment. These forms of child maltreatment also have serious consequences, similar to emotional and physical abuse, and future studies should also address them. Third, the respondents in the Wave 5 of CAPS were aged 21–29 and wages in the twenties probably are not the most informative of how these people will fare later in life, which is more interesting and instructive to the relationship of child maltreatment and economic consequences of adults. Longer-term studies should therefore also be conducted. Despite these limitations, this study contributes to the understanding of the economic loss and inequality caused by child maltreatment in a middle-income country, and may advance awareness of policy makers of the relationship between the national fiscus, and investments in intervention programs to reduce the prevalence of child maltreatment and help the victims better overcome the long-term negative effect of childhood violence.

Overall, our findings suggest a long-term economic consequence of child maltreatment – lower wages – that plays a key role in the economic burden and socioeconomic inequality of child maltreatment on developing countries. Developing countries cannot afford any loss to the economy, let alone one that results from a human rights violation, and it is clear that prevention and intervention programs in low- and middle-income countries like South Africa are urgently needed.

## Abbreviations

CAPS: Cape Area Panel Study
CTS: Conflict Tactics Scale
H2S: Heckman Two-Step Estimation
MLE: Maximum Likelihood Estimation
OLS: Ordinary Least Square
PoA: Programme of Action
USA: United States of America
USD: United States Dollar
